# Quantification and Identification of Post-Translational Modifications Using Modern Proteomics Approaches

**DOI:** 10.1007/978-1-0716-1024-4_16

**Published:** 2021

**Authors:** Anja Holtz, Nathan Basisty, Birgit Schilling

**Affiliations:** Buck Institute for Research on Aging, Novato, CA, USA.

**Keywords:** Acetylation, Post-translational modifications, Mass spectrometry, Data-independent acquisition, Quantification

## Abstract

Post-translational modifications (PTMs) occur dynamically, allowing cells to quickly respond to changes in the environment. Lysine residues can be targeted by several modifications including acylations (acetylation, succinylation, malonylation, glutarylation, and others), methylation, ubiquitination, and other modifications. One of the most efficient methods for the identification of post-translational modifications is utilizing immunoaffinity enrichment followed by high-resolution mass spectrometry. This workflow can be coupled with comprehensive data-independent acquisition (DIA) mass spectrometry to be a high-throughput, label-free PTM quantification approach. Below we describe a detailed protocol to process tissue by homogenization and proteolytically digest proteins, followed by immunoaffinity enrichment of lysine-acetylated peptides to identify and quantify relative changes of acetylation comparing different conditions.

## 1 Introduction

Proteomics techniques utilizing mass spectrometry (MS) have quickly become a preferred method to measure relative changes in protein abundance as well as significant changes in post-translational modifications (PTMs) (*see* also Chaps. 8, 13–15). Protein profiles consist of many unique proteoforms carrying different PTMs, which can exist simultaneously and change dynamically in response to environmental and other stimuli. One of the more common PTMs is lysine acetylation, which can regulate a multitude of physiological processes via changed protein-protein interactions, gene expression, and cellular location.

Post-translational modifications have been studied for decades; however, recent studies utilizing mass spectrometry have revolutionized the analysis of PTM profiles and identified thousands of novel PTM sites. Lysine acetylation has been reported to regulate many cellular pathways, specifically of mitochondrial proteins [1, 2]. However, many PTMs including lysine acetylation occur at a relatively low stoichiometry. Thus, enriching acetylated peptides via antibody-based affinity enrichment protocols have been established as a key methodology and workflow to measure the dynamics of the acetylome both in mammalian [3–5] and bacterial model systems [6–8]. Here, we present a detailed protocol to allow for a routine workflow of robust identification and quantification of lysine acetylation sites by affinity enrichment of PTM-containing peptides followed by mass spectrometric analysis. This will provide a standardized protocol for the study of lysine acetylation sites to streamline the identification and quantification of PTMs. This method provides a strategy to study PTMs with relatively low starting material, even with capabilities for multiplexing and enriching for multiple PTMs simultaneously [9] in an unbiased approach using label-free DIA-MS [10–12] (*see* also Chaps. 8, 22–24) in combination with multiple different software programs, such as Skyline and Spectronaut [13, 14] or others (*see* Chap. 31).

## 2 Materials

### 2.1 Tissue Lysis and Tryptic Protein Digestion

1. Tissue sample to be digested.
2. 2 mL Safe-Lock tubes.
3. Lysis buffer: 8 M (w/v) urea, 100 mM (w/v) triethylammonium bicarbonate (TEAB) pH 8.5, 1× protease inhibitor cocktail (Pierce), 5 μM (w/v) trichostatin A (TSA), 5 mM (w/v) nicotinamide, and 75 mM (w/v) sodium chloride (NaCl).
4. TissueLyser II and 5 mm stainless steel beads.
5. Bioruptor sonicator.
6. Bicinchoninic acid (BCA) protein assay.
7. Reducing reagent: 1 M (w/v) dithiothreitol (DTT), freshly prepared in deionized water.
8. Deionized water (referred to as H_2_O).
9. Alkylation reagent: 200 mM (w/v) iodoacetamide (IAA), freshly prepared in H_2_O.
10. Dilution buffer: 50 mM (w/v) triethylammonium bicarbonate (TEAB) in H_2_O.
11. Digestion enzyme: modified sequencing grade trypsin.

### 2.2 Desalting of Proteolytic/Tryptic Peptides After Digestion (Oasis/HLB)

1. Oasis HLB (hydrophilic-lipophilic balanced) 1 cc Vac cartridge, 30 mg sorbent per cartridge, 30 μm particle size.
2. Extraction manifold, 20-port vacuum manifold.
3. HPLC-MS grade acetonitrile (ACN) and water (H_2_O).
4. HPLC-MS grade formic acid (FA).
5. HLB Solvent A: 0.2% (v/v) FA in HPLC-MS grade H_2_O.
6. HLB Solvent B: 80% (v/v) ACN, 20% (v/v) of 0.2% FA in HPLC-MS grade H_2_O.

### 2.3 Anti-Acetyl Immunoaffinity Enrichment

1. PTMScan Acetyl-Lysine Motif [Ac-K] Immunoaffinity Beads.
2. PTMScan Immunoaffinity (IAP) Buffer: 50 mM MOPS, 10 mM Na_3_PO_4_, 50 mM NaCl in water at pH 7.2.
3. 1× phosphate-buffered saline (PBS): 0.01 M phosphate-buffered saline (0.0027 M KCl, 0.138 M NaCl) pH 7.4 at 25 °C.
4. Wide-bore 200 μL pipet tips.

### 2.4 Small-Scale Acetyl-Peptide Desalting Prior to MS Analysis

1. Empore Octadecyl (C18) 47 mm Extraction Disks (3 M).
2. 18-Gauge blunt-tipped needle and plunger.
3. VWR 200 μL low-binding pipet tips.
4. Multi SafeSeal Sorenson 0.65 mL microcentrifuge tubes.
5. Snap Cap Low Retention 1.5 mL and 2 mL graduated microcentrifuge tubes.
6. StageTip Solvent A: 0.2% FA in 99.8% HPLC-MS grade H_2_O (v/v).
7. StageTip Solvent B: 0.2% FA/50% (v/v) HPLC-MS grade ACN/49.8% HPLC-MS H_2_O (v/v/v).

### 2.5 Chromatography and Mass Spectrometry: Nanoflow HPLC-MS/MS

1. All HPLC-MS/MS buffers are “HPLC-MS grade.”
2. Mobile Phase A: 2% ACN/98% water/0.1% formic acid (v/v/v).
3. Mobile Phase B: 98% ACN/2% water/0.1% formic acid (v/v/v).
4. Nanoflow liquid chromatography: Ultra Plus nano-LC 2D HPLC (Eksigent) connected to a cHiPLC system (Eksigent) with a C18 pre-column chip (200 μm × 0.4 mm ChromXP C18-CL chip, 3 μm, 120 Å, SCIEX) and an analytical C18 column chip (75 μm × 15 cm ChromXP C18-CL chip, 3 μm, 120 Å).
5. Mass spectrometer: orthogonal quadrupole time-of-flight (QqTOF): TripleTOF 6600 system (SCIEX) or any other high-resolution mass spectrometry system.

## 3 Methods

### 3.1 Tissue Lysis

1. Harvest tissue of interest (here mouse liver), and take a portion ~50 mg wet weight to process immediately or freeze at −80 °C until ready.
2. Chill TissueLyser adapter sets to −20 °C for 1 h.
3. Prepare and label 2 mL Safe-Lock tubes on dry ice.
4. Add frozen tissue, and then add one stainless steel bead to each of the labeled tubes.
5. Add 500 μL ice-cold lysis buffer containing protease and deacetylase inhibitors (nicotinamide and TSA).
6. Vortex briefly, and spin to cover the whole tissue in lysis buffer. Add more lysis buffer if the tissue is not completely covered.
7. Place and balance tubes on chilled adapter sets. Homogenize with the TissueLyser II at 30 Hz twice for 3 min at 4 °C.
8. Remove bead with tweezer. Clean tweezer with deionized water and then HPLC-grade methanol, and dry between samples to prevent cross-contamination.
9. Spin briefly.
10. Sonicate on Bioruptor sonicator for ten cycles of 30 s on/30 s off at 4 °C.
11. Centrifuge homogenized tissue lysate for 15 min at 14,000 × *g*, 4 °C.
12. Transfer supernatant to new 1.5 mL tubes while avoiding the lipid layer above the cleared lysate and any pellet at the bottom of the tube.
13. Determine protein concentration using the BCA assay.

### 3.2 Tryptic Digestion

1. Remove an aliquot of lysate containing ~5 mg of soluble protein according to BCA assay (or more input material if available; *see* Note 1).
2. Add DTT to a final concentration of 4.5 mM to reduce disulfide bonds for 30 min at 37 °C with agitation.
3. Cool reduced lysate to room temperature (RT).
4. Add IAA to a final concentration of 10 mM to alkylate free thiols. Allow reaction to proceed for 30 min at RT in the dark.
5. Dilute reduced and alkylated proteins tenfold with 50 mM TEAB.
6. Add trypsin to initiate protein digestion (enzyme to protein ratio = 1:50, wt/wt) at 37 °C overnight with 1400 agitation.
7. Quench the digestion by adding FA to a final concentration of 1% (v/v) FA.
8. Remove undigested proteins and lipids by centrifugation for 10 min at RT with 1800 × *g*.
9. Desalt the supernatant containing peptides (*see* Subheading 3.3).

### 3.3 Desalt Tryptic Peptides with Oasis HLB Cartridges

1. Apply vacuum to the Oasis HLB 1 cc cartridges (30 mg sorbent; max. Binding capacity, 5 mg) using the vacuum extraction manifold, and condition the cartridges twice with 800 μL of organic HLB Solvent B.
2. Equilibrate cartridges three times with 800 μL of aqueous HLB Solvent A.
3. Load the acidified tryptic peptides onto the cartridge (here from 5 mg protein digest).
4. Wash the bound peptides with 800 μL of HLB Solvent A three times.
5. Place new, labeled 1.5 mL microcentrifuge tube into the vacuum manifold to collect eluting peptides.
6. Elute the peptides with the addition of 800 μL HLB Solvent B.
7. Elute once more with 400 μL HLB Solvent B.
8. Mix the elution with the vortexer, and then remove 2.4 μL (or 10 μg) for independent and parallel protein-level quantification (also *see* Note 2).
9. Concentrate/dry the eluted peptides completely using a SpeedVac (*see* Note 3).

### 3.4 Anti-Acetyl Immunoaffinity Enrichment

1. Resuspend the dried peptides in 1.4 mL cold IAP buffer, and mix by pipetting. Do not vortex.
2. Pipette 2 μL of the resuspended peptide solution onto litmus paper to check that the pH is neutral (between 7 and 8).
3. Centrifuge at 10,000 × *g* for 5 min at 4 °C. A small pellet may appear. Keep peptide solution on ice while preparing the antibody-bead conjugate (*see* Note 4).
4. Prepare the PTMScan Acetyl-Lysine antibody beads for peptide affinity enrichment by adding 1 mL cold 1× PBS to one tube of antibody-conjugated beads, and mix by pipetting. The ratio of PTMScan Acetyl-Lysine Motif antibody-conjugated beads to peptide starting material should be ¼ of a tube of antibody beads for 5 mg of peptides (*see* Note 1).
5. Transfer the slurry of antibody-conjugated beads to a new 1.5 mL microcentrifuge tube, and centrifuge at 2000 × *g* for 30 s at RT to prevent beads from sticking to the side of the tube.
6. Remove the PBS buffer by aspiration, and leave a small volume in the bottom to avoid disrupting the beads.
7. Wash the antibody beads with 1 mL cold 1× PBS, and centrifuge at 2000 × *g* for 30 s at RT. Remove the majority of PBS by aspiration.
8. Repeat the PBS wash step twice for a total of four PBS washes.
9. Resuspend the washed beads from one tube of PTMScan Acetyl-Lysine antibody in 440 μL PBS, and mix several times by pipetting with wide-bore 200 μL pipet tips.
10. Place four 100 μL aliquots of bead suspension into 1.5 mL microcentrifuge tubes. To ensure consistent bead quantities in the 100 μL aliquots, about 40 μL of beads will remain in the original tube (*see* Note 5).
11. Centrifuge the aliquoted beads at 2000 × *g* for 30 s at RT. Visually check that each tube has approximately the same quantity of antibody-conjugated beads.
12. Remove all 1× PBS by aspiration using a 0.2 mm flat gel loading pipet tip.
13. Transfer the resuspended peptides from **step 3** in Subheading 3.4 directly onto the prepared PTMScan Acetyl-Lysine Motif antibody-conjugated beads.
14. Incubate the peptides and antibody-conjugated bead mixture at 4 °C overnight on an end-over-end rotator or gentle mixer.
15. Centrifuge the peptide/bead mixtures at 2000 × *g* at 4 °C for 30 s.
16. Remove the supernatant, which contains unbound peptides, and save for further applications.
17. Wash the peptide-bound beads with 1 mL cold IAP buffer, and mix by inverting the tube five times.
18. Centrifuge at 2000 × *g*, 4 °C for 30 s. Remove the IAP wash solution by aspiration, and leave a small volume of IAP to avoid disrupting the beads.
19. Repeat the IAP wash once for a total of two washes.
20. Wash the peptide-bound beads with 1 mL ice-cold HPLC-MS water, and mix by inverting five times.
21. Centrifuge at 2000 × *g*, 4 °C for 30 s.
22. Remove the water wash solution by aspiration, and leave a small volume to avoid disrupting the beads.
23. Repeat the water wash twice for a total of three washes.
24. After the last water wash, centrifuge once more for 30 s at 2000 × *g*, 4 °C to collect any remaining volume to the bottom.
25. Aspirate the remaining water with 0.2mmgel loading flat pipet tip while avoiding the beads.
26. Add 55 μL 0.15% TFA in HPLC-MS water to the peptide-bound beads. Incubate at RT for 10 min. Mix by tapping the bottom of the tubes intermittently.
27. Centrifuge the mixture for 30 s at 2000 × *g*, RT.
28. Remove and transfer the eluted peptides with 0.2 mm gel loading flat pipet tip to a new, labeled 0.65 mL microcentrifuge tube.
29. Add 45 μL 0.15% TFA in HPLC-MS water to the peptide-bound beads, not the eluted peptides. Incubate the mixture at RT for 10 min with intermittent agitation by tapping the bottom of tubes.
30. Centrifuge the mixture for 30 s at 2000 × *g*, RT. Remove the second elution with a 0.2 mm gel loading flat pipet tip, and combine with the first elution.
31. Centrifuge the eluted peptides at 12,000 × *g* at RT for 5 min to pellet any beads that may have carried over. Store eluted peptides on ice for immediate desalting.

### 3.5 Small-Scale Acetyl-Peptide Desalting with C18 StageTips

1. Prepare the C18 StageTips for desalting as described by Rappsilber et al.: assemble a set of three disks (punched out with a 18-gauge needle from an Octadecyl C18 Extraction Disk membrane) in a low-binding 200 μL pipet tip, held together in a 0.65 mL Eppendorf tube with a hole in the bottom so the solvent can flow upon centrifugation into a 2 mL collection tube.
2. Condition the StageTip with 100 μL of 100% ACN by passing the supernatant through the assembly by centrifugation at 3000 × *g* for 1 min.
3. Wash the StageTip with 100 μL of Stage Tip Solvent B by centrifugation at 3000 × *g* for 1 min (*see* Note 6).
4. Equilibrate the StageTip with 100 μL of Stage Tip Solvent A by centrifugation at 3000 × *g* for 1.5 min. Repeat this step for a total of two equilibration washes.
5. Load the acidified immunoaffinity peptide elution from **step 31** onto the StageTip, and centrifuge at 3000 × *g* for 1.5 min.
6. Wash the peptides bound to the StageTip with 100 μL of Solvent A by centrifugation at 3000 × *g* for 1.5 min. Repeat this step for a total of two washes.
7. Elute the peptides with 50 μL of Stage Tip Solvent B into a new Eppendorf tube, and centrifuge at 3000 × *g* for 3 min to ensure all elution volume passes through.
8. Dry the peptide eluate completely using a SpeedVac.
9. Resuspend the peptides in an appropriate volume of mobile phase A of your LC-MS system, e.g., 7 μL of 2% ACN/98% water/0.1% formic acid (v/v/v), and add a retention time standard, such as 0.5 μL of indexed retention time standard (iRT from Biognosys or other standards).
10. Vortex the peptide solution for 10 min at 4 °C, and then centrifuge for 2 min at 12,000 × *g* and 4 °C.
11. Transfer the supernatant to an autosampler vial for nano LC-MS/MS (*see* Note 7).

### 3.6 Nanoflow LC-MS/MS Analysis

1. Samples are analyzed by reverse-phase HPLC-ESI-MS/MS using the Eksigent Ultra Plus nano-LC 2D HPLC system combined with a cHiPLC System, directly connected to a quadrupole time-of-flight TripleTOF 6600 mass spectrometer (SCIEX). Typically, mass resolution for precursor ion scans is ~45,000 (TripleTOF 6600), and fragment ion resolution was ~15,000 (“high sensitivity” product ion scan mode) (*see* Note 8). After injection, peptide mixtures are transferred onto a C18 pre-column chip and washed at 2 μL/min for 10 min with the Mobile Phase A (loading solvent). Subsequently, peptides are transferred to the analytical column ChromXP C18-CL chip and eluted at a flow rate of 300 nL/min typically with a 2–3 h gradient using aqueous and acetonitrile solvent buffers (Mobile Phases A and B).
2. *Data-dependent acquisitions (DDA)*. For spectral library building, initial data-dependent acquisitions (DDA) are carried out to obtain MS/MS spectra for the 30 most abundant precursor ions (100 ms accumulation time per MS/MS) following each survey MS1 scan (250 ms), yielding a total cycle time of 3.3 s.
3. *Data-independent acquisitions (DIA)*. For label-free relative quantification, all study samples are analyzed by data-independent acquisitions (DIA), using a 64-variable window SWATH acquisition strategy [10, 12]. Briefly, instead of the Q1 quadrupole transmitting a narrow mass range through to the collision cell, windows of variable width (5–90 *m*/*z*) are passed in incremental steps over the full mass range (*m*/*z* 400–1250). The cycle time of 3.2 s includes a 250 ms precursor ion scan followed by 45 ms accumulation time for each of the 64 DIA-SWATH segments.

### 3.7 Identification and Quantification of Acetylation Sites Using DDA and DIA

1. Mass spectrometric data from data-dependent acquisitions (DDA) is analyzed with the database search engine ProteinPilot 5.0 (SCIEX) using parameters such as trypsin digestion, cysteine alkylation set to iodoacetamide, and lysine acetylation, and in our case species *Mus musculus*, false discovery rates of 1% are used (*see* Note 9).
2. Using the database search engine results generated above, MS/MS spectral libraries are generated in Skyline-daily v19.1.1.248, an open-source data processing workspace for quantitative proteomics, DIA raw data files are imported into Skyline, and both MS1 precursor ion scans and MS2 fragment ion scans are extracted for all acetylated peptides present in the spectral libraries. In Skyline typically 6–10 MS2 fragment ions are extracted per acetylated peptide based on ranking from the corresponding MS/MS spectra in the spectral libraries, and fragment peak areas are summed per peptide.
3. Relative quantification of acetylation levels and comparisons of different conditions or strains (for example, knockout versus wild-type) can be performed directly in Skyline using integrated statistical algorithms. Statistical assessment of peak selection can be done within Skyline using mProphet, which was adjusted to specifics of DIA data. Alternatively, the corresponding extracted acetylation site peak areas can be exported and subjected to other open-source programs, such as mapDIA which is specialized for processing and statistical analysis of quantitative proteomics data from DIA-MS (*see* Note 8).

### 3.8 Anticipated Results

1. Depending on the experimental design, typically 1000–2000 acetylation sites or more can be identified and quantified from enrichment of 5 mg of protein lysate or 1 mg of isolated liver mitochondria.
2. Typically, 1–20 acetylation sites are detected per protein, but this varies clearly for each protein.
3. Workflow reproducibility can be assessed between replicates by using the coefficients of variations, CV, which we observe typically as <20%.
4. The affinity workflow using the PTMScan Acetyl-Lysine Motif [Ac-K] Immunoaffinity Beads to enrich for acetylated peptides typically yields high acetylation enrichment, with 50–70% of the peptides detected being acetylated.

## 4 Notes

1. We recommend a minimum of 5 mg whole protein input or 1 mg of protein isolated from liver mitochondria for the acetyl-lysine affinity enrichments. A maximum of 20 mg of protein from whole liver or 4 mg of protein from isolated liver mitochondria can be used following this protocol. Higher protein amounts are preferred for more robust results. Subsequently, the amount of antibody beads used should be changed according to the amount of protein used to maintain a high percentage of acetyl-peptide enrichment (50–70%). Ideally, an entire tube of PTMScan Acetyl-Lysine Motif antibody-conjugated beads should be used for 20 mg of peptides, and the amount of beads will change proportionately with the amount of protein used.
2. We normalize acetyl-peptide peak areas by dividing them by their corresponding protein-level areas. This allows us to determine which changes are truly based on the acetylation profile dynamics rather than changes in overall protein abundance. In **step 9** from Subheading 3.3 a small aliquot (100 μg) of digested and desalted protein was set aside to assess the total protein-level abundance changes for normalization.
3. Freeze the peptide resuspension solution quickly after drying, and keep it frozen throughout the drying process. This will allow for easy and consistent resuspension, resulting in a fluffy white/yellow powder after drying. If an oily film forms, the drying process was not performed optimally.
4. All steps during peptide and antibody-bead conjugate preparation should be done on ice. Centrifugation should be performed at 4 °C.
5. When creating aliquots of the antibody-bead conjugate, ensure there are approximately the same number of beads per aliquot by vortexing the beads immediately before removing aliquots.
6. Ensure the StageTips (packed with C18 disks) remain damp throughout all steps until after elution. Check the StageTips frequently during centrifugation and adjust the time accordingly, and remove StageTips which are flowing more quickly than others so they do not dry. Alternatively, C18 ZipTips can be used for desalting.
7. Even though there may be no visible material after centrifugation, handle samples delicately without agitation to avoid resuspending any small particulates.
8. Other high-resolution mass spectrometric systems from other instrument vendors will be able to similarly perform these label-free, high-resolution workflows.
9. To potentially increase the number of identified acetylated peptides, additional database search engines can be used after conversion of the raw data to mzXML format using msConvert from ProteoWizard.
